# Influence of on-road mobile monitoring design on ultrafine particle exposure models and cognitive health inferences

**DOI:** 10.1038/s41370-026-00845-y

**Published:** 2026-03-07

**Authors:** Magali N. Blanco, Annie Doubleday, Adam A. Szpiro, Julian D. Marshall, Paul K. Crane, Lianne Sheppard

**Affiliations:** 1 Department of Environmental and Occupational Health Sciences, University of Washington, Seattle, WA USA; 2 Department of Biostatistics, University of Washington, Seattle, WA USA; 3 Department of Civil & Environmental Engineering, University of Washington, Seattle, WA USA; 4 Department of Medicine, University of Washington, Seattle, WA USA

**Keywords:** Air pollution, Study designs, Exposure assessment, Epidemiology

## Abstract

**Background:**

On-road mobile monitoring is increasingly used to assess air pollution, but the implications of monitoring and analytic decisions on exposure prediction and health inferences remain unclear.

**Objective:**

This study evaluated the influence of on-road monitoring approaches in environmental epidemiology, specifically ultrafine particle (UFP) exposures and late-life cognitive function.

**Methods:**

We used data from a Seattle-based mobile monitoring campaign to develop a reference roadside UFP exposure model based on repeated measurements at 309 roadside locations and examine associations with cognitive function (Cognitive Abilities Screening Instrument—Item Response Theory [CASI-IRT]), in the Adult Changes in Thought cohort (*N* = 5283). To evaluate alternative designs, we subsampled on-road UFP measurements collected along 600 km of roadways, varying location visit frequencies, spatial balancing, and sampling times. New UFP models, some incorporating temporal and plume adjustments, were developed using universal kriging with partial least squares and used to estimate associations between UFP and CASI-IRT, after adjusting for age, year, sex, education, race, and socioeconomic status.

**Results:**

Using the reference exposure model in the primary health model, the mean baseline CASI-IRT score increased by 0.007 (95% CI: –0.013, 0.027) per 1900 pt/cm³ increment in PNC. Associations were similar but relatively attenuated for all on-road sampling designs. Route-based sampling (which accounted for logistical field constraints and spatiotemporal correlation in the data) and very short (4- vs 12-visit) campaigns produced more variable health estimates. Applying temporal and plume adjustments had a minimal impact on the inferential results.

**Significance:**

In analyses where no association was observed between UFP and cognitive function, the on-road monitoring design produced similar but slightly attenuated point estimates. Secondary analyses with a reduced health model, which indicated a statistically significant but potentially confounded association, suggest that on-road design—particularly monitoring beyond weekday business hours—may have greater implications in other contexts.

**Figure:**
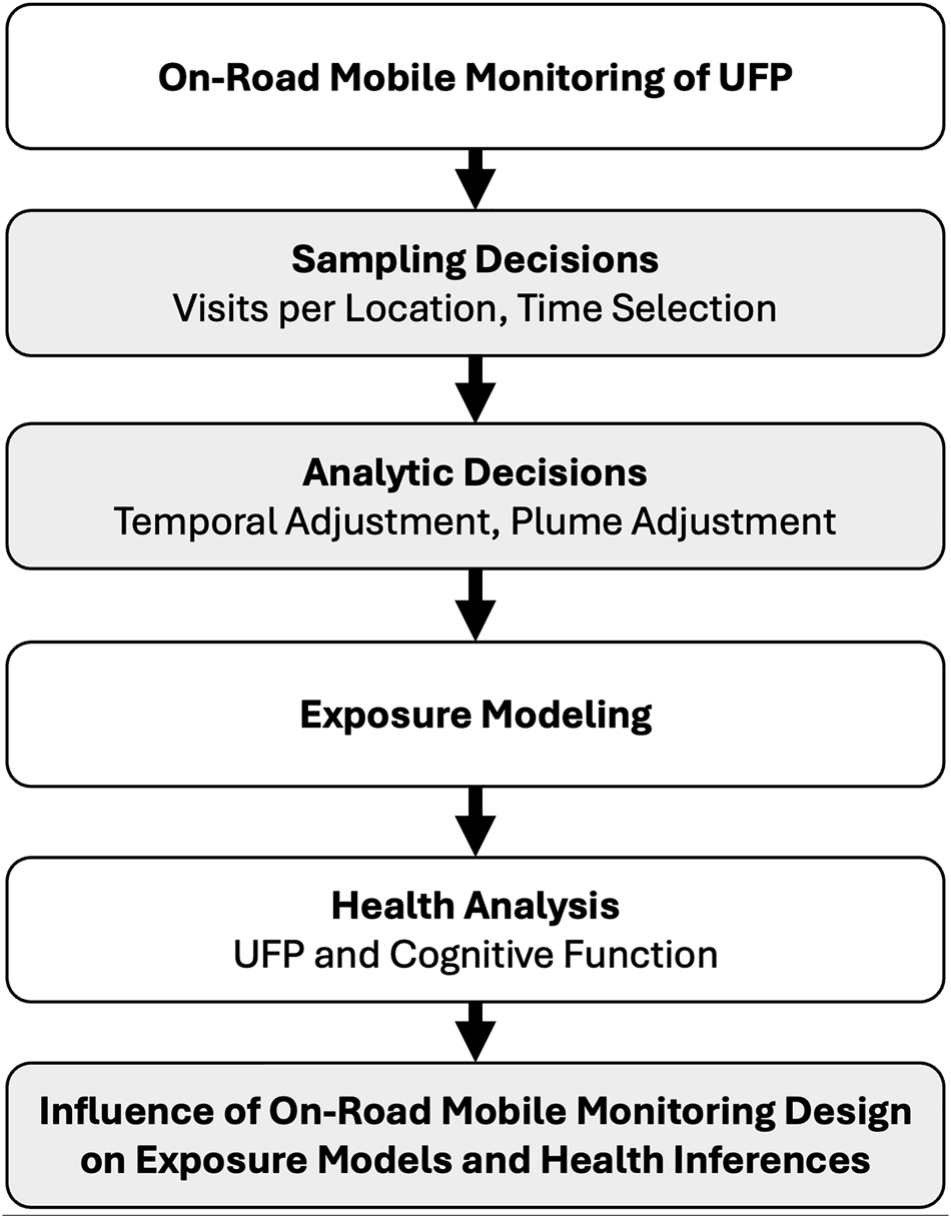
Tweetable Abstract: How does mobile monitoring design impact health studies? We assess UFP data and health measures to evaluate visit number, spatial balance, timing, and adjustments to assess their influence on exposure and health models.

**Impact statement:**

Mobile monitoring is increasingly used to develop air pollution exposure models, yet the influence of monitoring design on health inferences remains unclear. Using extensive ultrafine particle (UFP) data from a monitoring campaign and health measures from a long-standing cohort study, we assess how on-road campaigns can be designed for epidemiologic research. We evaluate the effects of visit number, spatial balance, time selection, temporal adjustment methods, and plume adjustments on exposure and health models, providing guidance for mobile monitoring design in air pollution health research.

## Introduction

Accurate air pollution exposure assessment is critical for environmental epidemiology. Mobile monitoring, the collection of repeated short-term samples from areas of interest, can be used to characterize poorly measured pollutants with high spatial resolution [1]. Traffic-related ultrafine particles (UFPs, ≤ 100 nm) are of growing interest given the accumulating evidence linking these to adverse cardiovascular, respiratory, and cognitive outcomes [2–5]. Still, the key design principles necessary to optimize on-road mobile monitoring for both exposure assessment and epidemiology remain unclear.

Several studies have evaluated how the number of repeat samples (site visits) impacts exposure estimates. Results may vary by pollutant, location, and averaging period, but generally, more measurements per location produce more precise concentration estimates. For example, in Oakland, CA, Messier et al. suggested that 4-8 visits per location for black carbon (BC) or nitrogen monoxide (NO) were sufficient [6], whereas Kerckhoffs et al. reported a number closer to 5-15 visits, depending on the modeling approach [7]. In Montreal, Canada, Hatzopoulou et al. found that 10-12 visits were necessary for UFP and nitrogen dioxide (NO_2_) [8]. Similarly, in Bengaluru, India, Upadhya et al. recommend 10 visits for UFP and BC [9].

Numerous gaps remain. We have previously demonstrated using fixed-site and stationary roadside monitoring data that limiting monitoring to weekday daytime hours leads to poorer exposure estimates and health inferences [10–12]. This has not been replicated for on-road monitoring campaigns, however, which measure pollutants at a greater number of unique locations and over shorter periods of time at any (less precise) location. Moreover, air pollution monitoring campaigns often lack spatially balanced measurements due to logistical constraints or intentional design choices, leading to variable visit frequencies. Additionally, while previous sampling studies have analyzed data at the segment level, allowing for independent sampling of neighboring segments, real-world campaigns often measure neighboring road segments consecutively along a route, resulting in the measurements for neighboring segments being temporally correlated.

There are also important analytic considerations for on-road campaigns that have not been evaluated. For instance, many campaigns apply temporal adjustments to temporally imbalanced data (e.g., from weekday business hours) to make the data more closely resemble longer-term averages of interest. A few studies have evaluated the effectiveness of these approaches using stationary or roadside monitoring data, finding that these can sometimes worsen exposure estimates [12, 13] and may not substantially improve health inferences [12]. Replicating this work for on-road campaigns, which offer limited temporal coverage at any given location, is critical.

Moreover, many studies [14–17], though not all [18], have reported higher air pollution concentrations from on-road campaigns when compared to fixed-site locations. On-road campaigns that measure in traffic or near to other vehicles have a higher likelihood of unintendedly over-sampling from the high concentrations found in exhaust plumes. Some investigations explicitly focus on on-road vehicle plumes, using on-road monitoring campaigns to follow specific vehicles and/or measure vehicle emission factors [9, 19–24]. Epidemiologic studies, however, are often more concerned with residential exposures, where on-road plume concentrations are generally less relevant. To decrease plume concentrations, methods such as plume detection [15], the use of stationary measurements [25], and the application of multi-pollutant measurements [14] have been developed. Most studies, however, have not implemented these approaches, and it remains unclear whether they necessarily improve exposure estimates and health inferences.

We leverage an extensive on-road and roadside mobile monitoring campaign [26] along with late-life cognitive function measures from the Adult Changes in Thought (ACT) cohort [27] to address key gaps in on-road mobile monitoring for environmental epidemiology. The primary objectives of this study were to evaluate the impact of on-road monitoring designs and analytic approaches on subsequent (1) exposure estimates and (2) epidemiologic inferences, specifically associations between UFP exposures and cognitive function. This study builds on prior work focused on stationary roadside monitoring but addresses distinct challenges specific to on-road mobile campaigns, including several that are not applicable to stationary designs. These include the use of route-based data collection, the elevated risk of spatial and temporal imbalance given the larger number of monitoring locations involved, and strategies to address potential limitations—such as temporal and plume adjustments—that are unique to on-road monitoring. These distinctions motivate a separate evaluation and provide a complementary contribution by highlighting key design principles for improving exposure assessment models and health inferences from on-road campaigns.

## Methods

### Study design

**Fig. 1:**
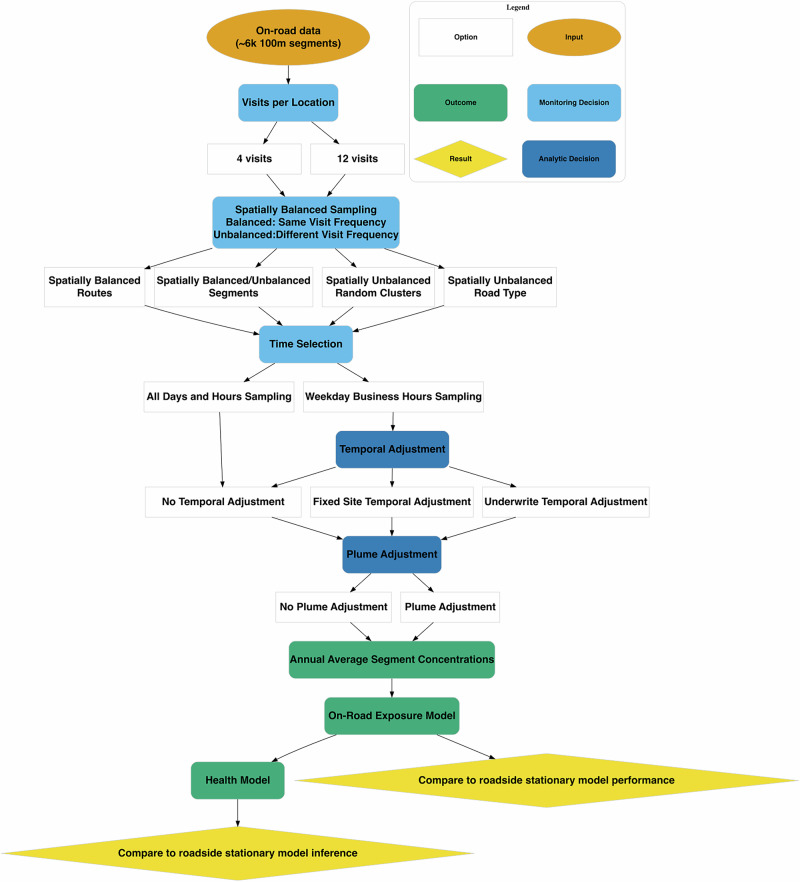
Flow diagram illustrating the process of evaluating on-road mobile monitoring design for epidemiology. The process includes monitoring decisions (such as the number of visits per location, spatial balance related to how locations are sampled, and sampling times), analytic decisions (including temporal and plume adjustments), exposure model development, and health analyses. We evaluate the robustness of exposure and health outcomes by comparing results to those from a roadside reference campaign. There are a total of 112 design combinations; each is repeated 30 times (campaigns) for a total of 3360 sampling campaigns in the main analyses.

We evaluated the influence of on-road monitoring design on UFP exposure models and subsequent associations with cognitive function in the ACT cohort. This study consisted of the following, as summarized in Fig. 1:

- Sampling on-road UFP measures following common monitoring approaches from an extensive monitoring campaign.
- Applying adjustments to account for temporally imbalanced and plume-impacted measurements.
- Developing UFP exposure prediction models with the sampled data.
- Evaluating the association between predicted UFP exposures and cognitive function in ACT.
- Comparing exposure and health estimates to those from a reference stationary roadside monitoring campaign.

### The ACT cohort and outcome ascertainment

We used cognitive function measures from the ACT cohort, as previously described [12]. In summary, ACT is a community-based, prospective cohort study investigating the aging brain in the greater Seattle area since 1994 [27]. The study randomly invites elderly individuals (65 + ) from Kaiser Permanente Washington to participate, assessing cognitive function at baseline using the Cognitive Abilities Screening Instrument (CASI), which evaluates attention, memory, language, and other cognitive functions [28]. Participants with high scores (≥86/100) are enrolled, while those with lower scores undergo further neuropsychological testing. Final CASI scores are derived using Item Response Theory (CASI-IRT) to enhance accuracy and account for missing items [29–31]. As of March 2020, the ACT study enrolled 5763 participants. This analysis included 5283 (92%) participants enrolled between 1994 and 2020, primarily excluding those who resided outside of the exposure monitoring region. Specifically, participants were included if they had a valid CASI-IRT score (5696, 99%), lived in the exposure monitoring region for at least 95% of the prior five years (5409, 94%), and had complete covariate information for adjustment (5283, 92%). Figure S1 in Blanco et al. 2025 further details participant retention.

### Mobile monitoring campaign

We analyzed UFP measurements from a previously described Seattle mobile monitoring campaign [14, 26]. Monitoring was conducted both on-road and at temporary (2-min) roadside stationary locations within a 1200 km^2^ area. Sampling occurred between March 2019 and March 2020, covering all four seasons, all days of the week, and most hours of the day (5 AM–11 PM). The overall campaign included repeat samples from approximately 600 unique roadway kilometers along nine fixed routes and 309 roadside locations representative of the cohort (see below for exclusions applied). Monitoring locations were selected to maximize spatial compatibility with the cohort. Specifically, the monitoring region was defined to include areas where most cohort members had historically resided. Individual roadside sites were then selected to minimize the distance between monitored and cohort locations while ensuring that the spatial distribution of monitoring sites reflected that of the cohort. Additional details are provided in Blanco, Gasset et al. [26]. This study focuses on total UFP (particle number concentration [PNC]) using the TSI P-TRAK 8525 instrument (size range: 20–1000 nm), which operated at a high measurement frequency of 1 Hz, enabling accurate PNC measurements during on-road monitoring. In contrast, prior work using stationary roadside measurements has employed the TSI NanoScan 3910, which captured smaller particles of interest (size range: 10–420 nm) but with a slower response rate of approximately 0.017 Hz (one measurement every 60 s), making it less suitable for on-road applications [12].

We divided the driving routes into 100 m segments and assigned all on-road measurements to the segment midpoint. We excluded A1 roads (interstates and highways with restricted access) to better represent residential exposures; segments with fewer than a median of five 1-s measurements per visit; segments with fewer than 23 repeat visits; and segments immediately before or after a roadside location, as these were not fully on-road measures. The 1-Hz PNC measurements were averaged over 10-s periods to align with other instruments, and the median PNC was calculated across all 10-s measures within each visit to a segment. Then for each segment, we winsorized these median values across visits: values below the 2.5th and above the 97.5th percentiles were set to those thresholds, to reduce the influence of extreme values. Overall, the median number of visits per road segment (*N* = 5874) was 28 (IQR: 27–28) [14].

We then adjusted the on-road data for high concentrations (“plumes”) using a previously described approach [14]. This method applies absolute principal component score (APCS) to identify on-road source components based on pollutants measured alongside UFP (BC, NO_2_, carbon dioxide [CO_2_], and other UFP measures). The contributions from these factors were then reduced to estimate adjusted UFP concentrations, with the final adjustment selected through an iterative process that leveraged data less likely to be plume-impacted, specifically roadside measures from the same campaign.

We used the resulting data to calculate the mean visit concentrations of each road segment to represent the annual average exposure estimate at that location.

Measures from roadside locations were processed similarly, with each of the 309 sites having approximately 28 (IQR: 28–28) winsorized median visit concentrations from the P-TRAK instrument [10]. These data were used to develop site-specific annual averages, which were treated as external reference measures for the on-road prediction models, as described below.

### Restricted air pollution mobile monitoring designs

We conducted hypothetical sampling campaigns to demonstrate how on-road mobile monitoring and analytic decisions influence exposure and health models. These included opportunistic designs with a limited number of visits per location and sampling restricted to typical working hours. We also evaluated the spatial imbalance that can arise when some locations receive more repeated measurements than others. Finally, we assessed whether known temporal imbalances and the presence of high-concentration plume events—potentially less representative of residential exposures—can be addressed analytically to improve the utility of on-road monitoring data for exposure assessment and health inference. Specifically, we sampled segment-level median UFP concentrations from drive-passes with replacement in the following process:

- **Visits per location:**
  - *4 visits*: We collected 4 visits per road segment location.
  - *12 visits*: We collected 12 visits per road segment location.

- **Spatially balanced sampling**: Visits were collected in spatially balanced or unbalanced approaches. Balanced approaches collected the same number of visits at all locations. Unbalanced approaches collected visit counts at each location that were guided by a lognormal distribution (e.g., mean: log 4, SD: log 2, range: 1–28) whereby some locations received more visits than others. We further considered spatial groupings by sampling at the route level, sampling at the segment level without grouping, sampling spatial clusters, or sampling based on road type, as described below.
  - *Spatially balanced routes*: All segments along a route were visited the same number of times and on the same day, thereby accounting for the temporal correlation inherent in field campaigns.
  - *Spatially balanced segments*: Each road segment was visited the same number of times, but we allowed for independent segment-level visits (e.g., on different days, not based on route), a common evaluation approach, but reflecting a design not logistically possible (i.e., the vehicle would have to jump around randomly in space).
  - *Spatially unbalanced segments*: Same as *spatially balanced segments*, but we allowed some segments to be visited more frequently than others.
  - *Spatially unbalanced random clusters*: Some segments were sampled more heavily by establishing spatial clusters, each composed of an average of 93 100-m segments (see SI Fig. S1). Segments within a cluster were sampled the same number of times and allowed to be independent (i.e., not based on route).
  - *Spatially unbalanced road type*: Road segments were sampled based on the predominant road type, with larger, more heavily trafficked roads receiving more visits.
- **Time selection**:
  - *All-day and hour sampling*: Visits were conducted during all days and hours.
  - *Weekday business hours sampling*: Visits were restricted to weekday business hours (9 AM–5 PM). For designs that sampled at the route (vs segment) level, more than 60% of the route was required to have been sampled during these hours.
- **Temporal adjustment**:
  - *No temporal adjustment*: No temporal adjustment was applied to the resulting data.
  - *Fixed-site temporal adjustment*: We followed a common approach of using a fixed-site background monitor to develop time-specific adjustment factors [32–35]. This method involves estimating time-specific adjustment factors based on PNC deviations at any given time (e.g., one hour in the study period) from the long-term average concentration at a background site. Since continuous PNC P-TRAK measures were not available for the entire study period, we simulated these from a fitted model of collocated P-TRAK PNC and NO_2_ measures. We applied the resulting adjustment factors to the temporally unbalanced data. This approach assumes that temporal fluctuations in PNC concentrations at the background site reflect the temporal fluctuations of each monitored site, and that the adjusted concentrations better represent the long-term exposure of interest for epidemiologic inference. Additional methodological details are presented in Blanco et al. 2025 and the corresponding supplemental material.
  - *Underwrite temporal adjustment*: We simulated a low concentration “background” site using the measures collected during a given campaign following an approach adapted from Hankey & Marshall [36, 37]. Specifically, for each campaign, we used the collected 1-s PNC data to estimate background levels by calculating the first percentile of three-hour rolling averages. We then computed both hourly and long-term (~1 year) average background PNC values. Hourly temporal adjustments were estimated as the difference between hourly and long-term background PNC, and these adjustments were applied to the mobile monitoring data. See Note S1 for additional details on this approach.
- **Plume adjustment**:
  - *No plume adjustment*: We used the raw measures.
  - *Plume adjustment*: We applied the plume adjustment detailed by Doubleday et al. [11] and summarized in the Methods. This method reduces the influence of transient, high on-road concentrations and produces adjusted concentrations that may be more representative of longer-term ambient conditions under the assumption that unusual spikes in pollutant concentrations may not reflect typical residential exposures.

In sensitivity analyses, we visited routes more times (20 visits) and used larger spatial clusters (Fig. S1).

For each campaign, we calculated the annual average concentration for each sampled road segment. We repeated this process 30 times to increase robustness, resulting in a total of 3360 sampling campaigns (112 designs x 30 campaigns each) in the main analyses. The annual averages from each campaign were used to develop an exposure prediction model, which was subsequently applied to evaluate the association between UFPs and cognitive function, as described below.

### Air pollution exposure assessment

We developed exposure prediction models for the annual average roadside PNC reference model (*n* = 309), as previously described [12], as well as from each on-road sampling campaign. Models for PNC were developed using universal kriging—partial least squares (UK-PLS) and hundreds of geographic covariates predictive of TRAP (e.g., land use, roadway proximity, population density) [10].

We evaluated each on-road exposure model by predicting PNC at out-of-sample stationary roadside sites and comparing these predictions against the observed annual averages for those locations. We employed this approach because we hypothesized that the short-term stationary roadside measurements were more representative of residential exposures (thus more suited for epidemiologic application) than the on-road measures because: (1) they were physically further from the center of the road and closer to residential facades; (2) they may be less impacted by on-road traffic plumes; (3) the measurement duration per location is slightly longer than for on-road locations (e.g., minutes vs. seconds), thus potentially resulting in more stable long-term averages; and (4) measures were collected and linked to exact (stationary) locations rather than the midpoints of longer 100 m road segments. We evaluated model performance using mean-square error (MSE) -based R^2^ (${R}_{{MSE}}^{2}$), which evaluates whether pairs of observations and predictions are identical, as well as using more common regression-based R^2^ (${R}_{{reg}}^{2}$), which evaluates whether pairs are linearly associated.

We used each campaign exposure model to predict time-weighted average PNC exposures for each ACT participant at baseline based on their prior five-year residential history. Given the limited availability of PNC data, this analysis assumed that the 2019–2020 PNC exposure surfaces reflected longer-term exposures and remained stable over time [38–44] and were therefore applicable to the cohort. Our prior work evaluating air pollution and cognitive function in the ACT cohort yielded similar results when analyses were restricted to more recent enrollment periods closer to 2019 [12]. This is further addressed in the Discussion.

### Inferential analyses

We used data from ACT (described in 2.2 The ACT Cohort and Outcome Ascertainment) to evaluate the association between baseline cognitive function (CASI-IRT) and predicted PNC exposure from each exposure model using the following:

1 $${Y}_{j}={{\rm{\alpha }}}+{\beta }_{m}{\hat{X}}_{j,m}+{\sum }_{i}{\delta }_{m,i}{W}_{j,i}+{\epsilon }_{j}$$

where ${Y}_{j}$ is the baseline CASI-IRT for participant $j$; $\alpha$ is the modeled intercept; ${\hat{X}}_{j,m}$ is the predicted five-year average air pollution exposure prior to baseline for participant $j$ from exposure model $m$; ${\beta }_{m}$ is the estimated association between air pollution and CASI-IRT and the main parameter of interest; and ${W}_{j,i}$ and ${\hat{\delta }}_{m,i}$ are indexed by adjustment variable $i$ with ${\hat{\delta }}_{m,i}$ representing the respective estimated coefficient. Reduced models adjusted for participant age, calendar year, sex, and education. Primary models further adjusted for race and socioeconomic status based on the Neighborhood Disadvantage Index [45] of each participant’s longest-lived address at or prior to baseline. We used robust standard errors (sandwich estimator) to improve the reliability of our inferences [46–48] and a p-value threshold of 0.05 to determine statistical significance.

We compared the estimated health association (${\hat{\beta }}_{m}$) from applying each on-road exposure model to the association estimated from using the reference roadside exposure model.

We conducted all analyses in R (v. 4.2.2) [49].

## Results

### Cohort characteristics

ACT participants had an average age of 74 years (SD: 6), with a slight majority (58%) being female. Over half of the participants held a high school or college degree, and the average CASI-IRT score was 0.33 (SD: 0.71). There were no meaningful demographic differences across UFP exposure levels, although participants in the highest PNC exposure tertile (as determined by the roadside reference PNC model) had slightly lower CASI-IRT scores compared to those in the lower tertiles. Additional cohort characteristics are reported in Blanco et al. [12].

### Model performances and exposure assessment

**Fig. 2:**
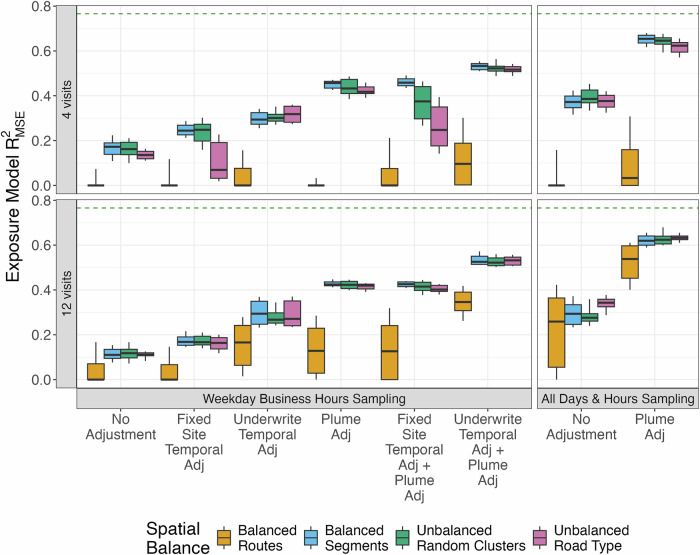
Out-of-sample PNC (pt/cm^3^) exposure model performances for on-road campaigns (*N* = 30 campaigns per combination, represented by boxplots). The top plot shows 4-visit campaigns, while the bottom plot shows 12-visit campaigns. ${R}_{{MSE}}^{2}$ is based on the comparison of the predicted PNC at 309 roadside locations to the annual average site estimates at those locations from stationary measures. The dashed green line represents the ${R}_{{MSE}}^{2}$ from the reference roadside exposure model, which is 0.77. Boxes show the median and IQR, and whiskers indicate the 10th and 90th percentiles. Spatially balanced approaches collected an equal number of visits across all locations, whereas unbalanced approaches resulted in uneven sampling, with some locations visited more often than others (e.g., those near certain road types or within spatial clusters). Details on spatial balance are provided in Section 2.4, Restricted Air Pollution Mobile Monitoring Designs.

The reference roadside PNC model had a cross-validated ${R}_{{MSE}}^{2}$ of 0.77. Campaigns that collected measurements during all hours of the day and applied a plume adjustment performed the best, while business hours campaigns generally performed the worst (Fig. 2). Temporal adjustments slightly improved the performance of business hours designs, although they introduced more variability in the results across campaigns, and the resulting improvement was smaller than plume adjustment. Temporally- and plume-adjusted business hours campaigns resulted in exposure models that performed similarly to unadjusted all-hours designs. Sampling designs that accounted for route structure—intended to reflect the temporal correlation observed in realized campaigns—consistently performed worse compared to random segment-based sampling, which involves random spatial jumps that are physically unrealistic for actual mobile sampling. In fact, compared to analyses that sampled 4 visits per location at the segment level, analyses that sampled at the route level produced much more variable, worse performances, even when locations are visited 20 times (5 times as much; Fig. S4). Moreover, designs that considered route also benefited from all-hours sampling and the application of temporal and plume adjustments. We found similar results when we used larger spatial clusters to conduct spatially unbalanced sampling (Fig. S5).

As expected, exposure model performances reported using ${R}_{{reg}}^{2}$ were consistently higher than ${R}_{{MSE}}^{2}$ (Fig. S6). Moreover, ${R}_{{reg}}^{2}$ struggled to differentiate performance differences between designs. One exception was the route-based sampling design, which consistently showed poorer performance compared to other designs.

In secondary analyses, we found that fixed-site temporal adjustment sometimes, but not always, improved ${R}_{{MSE}}^{2}$ (Fig. S7). Underwrite temporal adjustments slightly improved ${R}_{{MSE}}^{2}$.

Cohort predictions from the roadside reference UFP model had a median (IQR) of 7008 (6212–7995). On-road sampling campaigns generated PNC predictions that were slightly more elevated, with plume adjustments reducing these inflated predictions (Fig. S8).

### Inferential analyses

**Fig. 3:**
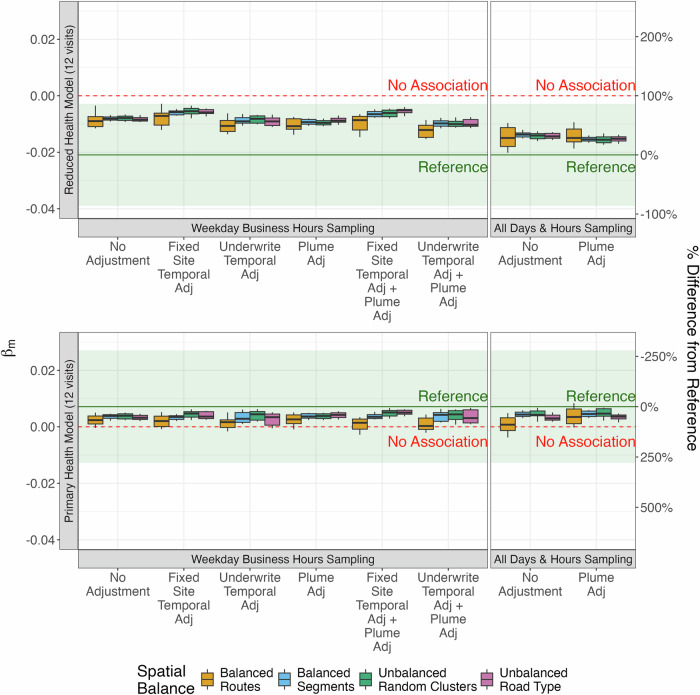
Estimated association between cognitive function (CASI-IRT) and PNC exposure (per 1900 pt/cm³) for 12-visit campaigns. The top plot shows the reduced health model, while the bottom plot shows the primary health model. The dashed green line and colored area represent the reference health estimate and 95% CI from the roadside exposure model. The dashed red line indicates no association. Boxes show the median and IQR; whiskers illustrate the 10th and 90th percentiles of on-road campaign point estimates. Spatially balanced approaches collected an equal number of visits across all locations, whereas unbalanced approaches resulted in uneven sampling, with some locations visited more often than others (e.g., those near certain road types or within spatial clusters). Details on spatial balance are provided in Section 2.4, Restricted Air Pollution Mobile Monitoring Designs.

In the reduced health model, the adjusted mean baseline CASI-IRT score decreased by 0.021 (95% CI: –0.039, –0.003) per 1900 pt/cm³ increment in PNC, as determined by the reference exposure model. Although associations were slightly attenuated across all on-road designs, the all-day and hourly sampling designs produced the most consistent results, particularly in plume-adjusted campaigns, which produced a median point estimate of –0.015 (IQR: –0.016, –0.014) for the balanced segment designs (Fig. 3). Weekday business hours sampling campaigns resulted in more attenuated health associations (e.g., unadjusted balanced segment design median point estimate –0.008, IQR: –0.009, –0.007). Route-based designs demonstrated greater variability in health estimates (e.g., unadjusted all-day and hour-balanced route design median point estimate –0.015, IQR: –0.018, –0.011). Compared to unadjusted weekday business hours designs, underwrite and plume-adjusted campaigns produced health estimates that were marginally closer to the reference model (e.g., balanced segment design median point estimate –0.010, IQR: –0.011, –0.009). In contrast, fixed-site temporally adjusted designs yielded slightly more attenuated estimates (e.g., balanced segment design median point estimate –0.006, IQR: –0.007, –0.005). Fig. S9 provides point estimates and corresponding 95% confidence intervals for selected designs. Point estimates fluctuate around the reference estimate, while their CIs generally show consistent uncertainty (i.e., similar standard errors).

In the primary health model, the adjusted mean baseline CASI-IRT score increased by 0.007 (95% CI: –0.013, 0.027) per 1900 pt/cm³ increment in PNC, reflecting associations that were consistent with both adverse and protective effects and not statistically significant. As before, associations were slightly attenuated across all on-road designs, with route-based designs continuing to show greater variability in estimates (e.g., unadjusted all-day and hour-balanced route design median point estimate 0.001, IQR: –0.001, 0.003; vs. similar balanced segment design median point estimate 0.004, IQR: 0.004, 0.005). However, there was substantial overlap in health associations across all designs, making the benefit of specific monitoring designs less clear. Figure S10 provides point estimates and corresponding 95% confidence intervals for selected designs.

We observed similar patterns when we evaluated campaigns with 4 and 12 visits, with 4-visit designs producing more variable results (Fig. S11).

## Discussion

We leveraged an extensive mobile monitoring campaign and a long-standing cohort study to gain insight into the impact of common on-road monitoring and analytic decisions on both exposure estimates and health inferences.

Using this reference roadside exposure model in a primary health model, we observed no strong evidence of an association between CASI-IRT score and PNC exposure. Interestingly, this association was slightly attenuated across all on-road monitoring designs, potentially due to lower exposure model performances compared to the reference roadside campaigns (Fig. 2). There was also considerable overlap in the health estimates from all on-road designs, with route-based sampling designs and, as expected, shorter 4 (vs. 12) visit campaigns producing more variable results (Fig. S11), potentially due to the large decreases in temporal representation—which may be important for capturing long-term averages—resulting from these designs. Campaigns that collected spatially and temporally balanced samples had a relatively small impact in this context; however, this was possibly because health estimates were not statistically significant.

It’s notable that, while we aimed to evaluate monitoring design in an analysis with a statistically significant association, we observed this only in reduced health models potentially influenced by residual confounding. In these analyses, all-hours sampling designs with more robust exposure assessment models produced results most consistent with reference health estimates (Fig. 2 and Fig. 3). More restricted weekday business hours sampling, which generally produced less accurate annual average estimates and predicted exposures, produced more attenuated results. Monitoring designs may thus be more critical in contexts when statistically significant associations are present.

Interestingly, while exposure models improved with temporal adjustment, health associations were minimally impacted by temporal adjustments. We previously observed similar results using stationary roadside mobile monitoring data [12]. In this work, we found that collecting temporally balanced visits may be important in some contexts, and that temporally adjusted measurements yield inconsistent improvements in health inferences [10, 12]. Similar findings have been reported in other studies using fixed-site monitoring data, where common temporal adjustment approaches were found to reduce the accuracy of air pollution exposure predictions and even worsen predictions [13]. Moreover, the differing exposure model performances between the fixed-site and underwrite adjustment approaches likely reflect the inherent strengths and limitations of each method. For instance, the fixed-site adjustment approach incorporated measurements across all hours during the study period, including unmonitored times (i.e., overnight), but was constrained to the temporal trend of a single location. Conversely, the underwrite approach leveraged data from all locations within a campaign to provide richer spatial information, but it was limited to the monitoring hours, reducing its ability to correct for non-sampled times. Compared to fixed site adjustments, underwrite adjustments were generally smaller (e.g., IQR: –1087 to 1422 vs. –1406 to 2145 pt/cm^3^; Table S1). The better performance of the underwrite approach (Fig. S7) may be explained by its ability to capture the spatial variability of UFPs more comprehensively, while requiring less extreme adjustments. It is notable that the fixed-site adjustment approach used in this study was based on a simulated UFP monitoring site rather than actual UFP observations [12]. UFP field monitoring campaigns will face similar constraints given the limited availability of continuous UFP measurements. In cases where exposure predictions improved, temporal adjustments might have reduced Berkson-like measurement error by better aligning the adjusted annual averages with the true reference values. Conversely, in cases where exposure predictions did not improve, the temporal adjustment might have introduced more classical-like measurement error than the reduction in Berkson-like measurement error, ultimately decreasing overall prediction accuracy [50, 51]. Given that these post-hoc adjustments can lead to inconsistent results, conducting temporally balanced sampling increases the likelihood that exposure estimates will be more accurate.

Similar to temporal adjustments, plume adjustment improved the ability of on-road exposure models to predict roadside concentrations—likely due to down-weighting measurements affected by transient spike on-road concentrations—but it did not influence health inferences. This was true both in the primary analyses, where we observed no statistically significant association between UFPs and cognitive function, and in reduced health models, where the association was statistically significant. Plume adjustment may thus be less critical than other monitoring design features—most notably, the inclusion of measurements beyond weekday business hours, which improved health estimates in reduced models. The collection of roadside measurements may also be valuable, given the minimal impact of the plume adjustment applied in this study. We evaluated one approach that leverages both stationary and multi-pollutant measures [14], but other approaches have been developed to address plumes [15, 25] and could be explored in future work.

Our findings also suggest that route-based sampling should be carefully considered when evaluating reduced sampling campaigns to avoid underestimating the number of visits needed for robust exposure assessment. In practice, adjacent road segments are generally sampled during similar time periods, significantly diminishing the overall temporal coverage that a campaign can achieve. Ignoring this temporal correlation can lead to overly optimistic results, falsely suggesting that far fewer visits are sufficient to produce reliable exposure estimates and health inferences. This may be particularly true when evaluating results with geostatistical models that borrow information from nearby locations.

Interestingly, collecting measurements with equal frequency across locations (i.e., spatial balance) appeared to have the least impact, with minimal effects on both exposure and health models. This is practically helpful for mobile monitoring campaigns, where achieving spatial balance can be logistically difficult—particularly for hard-to-reach locations. In prior work, however, we evaluated more extreme scenarios of spatial imbalance at roadside locations and found that exposure model performance worsened when highly variable sites were sampled only a few times (~2 visits), compared to more stable sites with more frequent sampling (~22 visits) [12]. While this had minimal impact on health inferences on average, it was associated with greater variability across campaigns. Taken together, some degree of spatial imbalance in mobile monitoring may be acceptable; however, it is possible that more extreme imbalances, if evaluated, could result in less stable health estimates.

We found that ${R}_{{reg}}^{2}$ consistently reported much higher exposure model performances and struggled to differentiate between most designs when compared to ${R}_{{MSE}}^{2}$. While ${R}_{{reg}}^{2}$ was able to detect large performance differences due to restricted sampling hours and route-based sampling approaches, alternative performance metrics like ${R}_{{MSE}}^{2}$ may provide more accurate assessments of the key interest: whether observations and predictions are identical, and not just linearly related. Additionally, it’s important to note that we used roadside reference site concentrations, which are more stable than on-road measurements (as discussed in the Methods and below), to evaluate exposure predictions. The choice of reference measures will also impact the results [52].

There are many other monitoring designs and analytic decisions that may be important to consider in future work. Future research may benefit from exploring other sampling strategies, such as approaches that allow for reduced sampling at locations where exposure levels can be more easily predicted or those that evaluate spatially unbalanced route-based sampling. These considerations could further enhance the efficiency and accuracy of mobile monitoring campaigns. Still, findings from our prior work with roadside monitoring campaigns may also be applicable to on-road campaigns [10, 12]. For instance, we previously reported that campaigns with samples collected across 3-4 seasons produced the most robust annual average exposure models and subsequent health inferences. Moreover, although this study focused on UFPs, a pollutant commonly measured with mobile monitoring, we previously observed similar findings for other pollutants, including BC, NO₂, fine particulate matter (PM_2.5_), and CO₂ when evaluating the number of visits and campaign durations required to produce accurate exposure models [10]. It would be valuable for future work to evaluate these and other design considerations for UFPs, as well as other pollutants across different geographic regions and health outcomes. It is possible that different design approaches may become more important in certain settings, depending on pollutant characteristics and the strength of the health association. Another consideration is that results may vary somewhat when using alternative approaches to exposure modeling (e.g., machine learning). We expect that our conclusions based on UK-PLS will generalize to other well-performing models, whereas lower-performing approaches (e.g., approaches with limited covariates, or models that have been over-fit) may introduce greater exposure error, potentially obscuring differences in health effect estimates attributable to monitoring design. Finally, while this study focuses on the implications of monitoring design for exposure assessment and health inference, our prior work focuses on a related and practical evaluation of the trade-offs between design, exposure performance, and costs [53].

This study had several limitations. We observed that results varied based not just on the monitoring design and analytic decision, but on the exact health analysis (i.e., primary vs. reduced health models), underscoring the importance of reproducing this work in other settings. Additionally, we used mobile monitoring measurements from 2019 to 2020 in our health analyses under the assumption that exposure surfaces had not changed over time [38–44]. This approach likely introduced exposure measurement error due to temporal misalignment, particularly for earlier years. While our prior work has shown similar findings when restricting analyses to more recent periods [12], future studies should replicate these analyses as longer-term UFP exposure models become available. Furthermore, the plume adjustment applied in this study was derived from the original mobile monitoring campaign, which included a greater number of on-road and roadside measurements than the campaigns sampled in this study. This approach may be overly optimistic, as it assumes that this extensive data will be available. Still, to our knowledge, this is the first study to evaluate the impact of plume adjustment on health inference. Finally, it is possible that design considerations may differ across regions and pollutants (see previous paragraph), as source types, ambient conditions, and dispersion patterns can vary, making some aspects more relevant.

A strength of this study is the use of real data from the long-standing ACT cohort. These data naturally incorporate complexities that might not be captured in a simulation study, thereby enhancing the relevance of our findings. Additionally, we utilized spatially- and temporally balanced roadside measurements to evaluate on-road model performances, which are not always available in practice. Comparing long-term model predictions against more restricted, unbalanced measurements from sampling campaigns can yield unclear results for some designs, particularly when predictions are compared against unstable estimates [10, 52].

In closing, on-road campaigns that collect data using temporally balanced approaches can be used to improve exposure assessment models. Plume and temporal adjustments can sometimes improve exposure models from temporally restricted campaigns, but these may minimally improve subsequent health inferences. Increasing the number of visits per location increases temporal representation and generally leads to more consistent findings. It is important to consider the temporal correlation inherent in realized on-road field campaigns when assessing the impacts of reduced sampling approaches. In terms of health inference implications, we found that on-road monitoring campaigns generally produced attenuated health estimates. In contexts where no association was observed between UFPs and cognition, monitoring design had minimal impact. Monitoring design may have a greater influence in contexts where robust, statistically significant health associations are observed, although more research is needed in this area.

## Supplementary information

Supplementary Information

## Data Availability

The analytic code for these analyses is available on GitHub (https://github.com/magali17/hei_aim3a). A Zenodo repository currently hosts relevant data from the mobile monitoring campaign (https://zenodo.org/records/13761282). The authors plan to add the on-road monitoring data to this repository following the completion of related ongoing analyses.
