# Supplementary material for: Influence of on-road mobile monitoring design on ultrafine particle exposure models and cognitive health inferences
Source: J Expo Sci Environ Epidemiol. 2026 Mar 7;36(3):575–84. doi: 10.1038/s41370-026-00845-y (PMC13143814; doi:10.1038/s41370-026-00845-y)
Supplement: Supplementary file 1 — Supplementary Information [file 41370_2026_845_MOESM1_ESM.pdf]

Supplemental Material

Influence of on-road mobile monitoring design on ultrafine particle exposure models and cognitive health inferences

Magali N. Blanco,[1] Annie Doubleday,[1] Adam A. Szpiro,[2] Julian D. Marshall,[3] Paul K. Crane,[4] Lianne Sheppard[1,2]

[1]Department of Environmental and Occupational Health Sciences, University of Washington, Seattle, WA USA
[2]Department of Biostatistics, University of Washington, Seattle, WA USA
[3]Department of Civil & Environmental Engineering, University of Washington, Seattle, WA USA
[4]Department of Medicine, University of Washington, Seattle, WA, USA

Table of Contents

1 METHODS **4**

2 RESULTS **8**

List of Figures

Figure S1. Spatial clusters used to conduct the unbalanced sampling designs. The main clusters (left) include an average of 93 100 m segments per cluster. Sensitivity clusters have approximately three times as many segments 4

Figure S2. Example time series of measured and simulated background PNC for two sampling days. One-second P-TRAK field measurements are shown in purple. Background PNC is calculated as the 1st percentile of three-hour rolling windows (green); these values are used to estimate hourly PNC (red) and long-term average PNC (teal). The primary analysis focuses on non-highway measurements, while sensitivity analyses incorporate all road types, represented by different line styles 6

Figure S3. Example of temporal adjustments in sensitivity analyses evaluating different percentiles (1st to 10th) and time windows (1 to 3 hours) to define underwrite functions. (a) The top two rows show estimated hourly background concentrations and long-term averages (horizontal lines) for different sampling days. (b) The bottom two rows show the corresponding temporal adjustments. Different road types ("all road types" vs. "no highways") are represented by different line types. Despite variations in time windows, percentiles, and road types, results are highly consistent across all scenarios. 7

Figure S4. Out-of-sample PNC (pt/cm³) exposure model performances for on-road campaigns (N=30 campaigns per combination - i.e., boxplot) when sampling at the segment- vs. route-level. Compared to analyses that sample 4 visits per location at the segment level, analyses that consider logistical field constraints and sample entire routes generally perform worse, even when locations are visited 20 times (5 times as much). $RMSE2$ is based on a comparison of the predicted PNC at 309 roadside locations to the annual average site estimates at those locations from stationary measures. The boxes illustrate the median and IQR, while the whiskers extend to the 10th and 90th percentiles. The dashed line shows the performance of the stationary roadside model. 8

Figure S5. Out-of-sample PNC (pt/cm³) exposure model performances for on-road campaigns (N=30 campaigns per combination - i.e., boxplot) based on spatial clusters used in the main analysis and alternative, larger clusters. Results are for campaigns with 12 visits per location. $RMSE2$ is based on a comparison of the predicted PNC at 309 roadside locations to the annual average site estimates at those locations from stationary measures. The boxes illustrate the median and IQR, while the whiskers extend to the 10th and 90th percentiles. The dashed line shows the performance of the stationary roadside model. 9

Figure S6. Out-of-sample PNC (pt/cm³) exposure model performances for on-road campaigns (N=30 campaigns per combination - i.e., boxplot) evaluated using $Rreg2$ for 12-visit campaigns. $Rreg2$ is based on a comparison of the predicted PNC at 309 stationary locations and the annual average site estimate based on stationary measures. The boxes illustrate the median and IQR, while the whiskers extend to the 10th and 90th percentiles. The dashed line shows the performance of the stationary roadside model for respective R^2, which is the same for $RMSE2$ and $Rreg2$. 10

Figure S7. Comparison of $RMSE2$ for a subset of 12-visit business hours sampling designs with and without temporal adjustment. The diagonal dashed line is the 1-1 line; values above this line indicate better resulting $RMSE2$ values when compared to unadjusted campaigns 11

Figure S8. Predicted five-year average PNC exposure for ACT participants across 30 sampling campaigns for each design (i.e., boxplot). The green dashed line and area represent the median and IQR of cross-validated PNC predictions from the reference all-data stationary exposure model. The boxes illustrate the median and IQR, while the whiskers extend to the 10th and 90th percentiles. 12

Figure S9. Point estimates and 95% confidence intervals (CI) from reduced health models of the adjusted association between PNC (1,900 pt/cm³) and CASI-IRT for selected campaigns. The green line and shaded area represent the point estimate and 95% CI from the roadside all-data exposure model, while the red line indicates no association. 13

Figure S10. Point estimates and 95% confidence intervals (CI) from primary health models of the adjusted association between PNC (1,900 pt/cm³) and CASI-IRT for selected campaigns. The green line and shaded area represent the point estimate and 95% CI from the roadside all-data exposure model, while the red line indicates no association. 14
Figure S11. Estimated association between cognitive function (CASI-IRT) and PNC exposure (per 1,900 pt/cm³) for 4-visit campaigns. The dashed green line and colored area represent the reference health estimate and 95% CI from the roadside exposure model. The dashed red line indicates no association. Boxes show the median and IQR; whiskers illustrate the 10th and 90th percentiles of on-road campaign point estimates. 15

List of Tables

Table S1. Distribution of hourly P-TRAK PNC (pt/cm³) temporal adjustments applied to adjust business hours campaign visits. 8

List of Notes

Note S1. Summary of temporal adjustment using an underwrite function approach 5

1 Methods

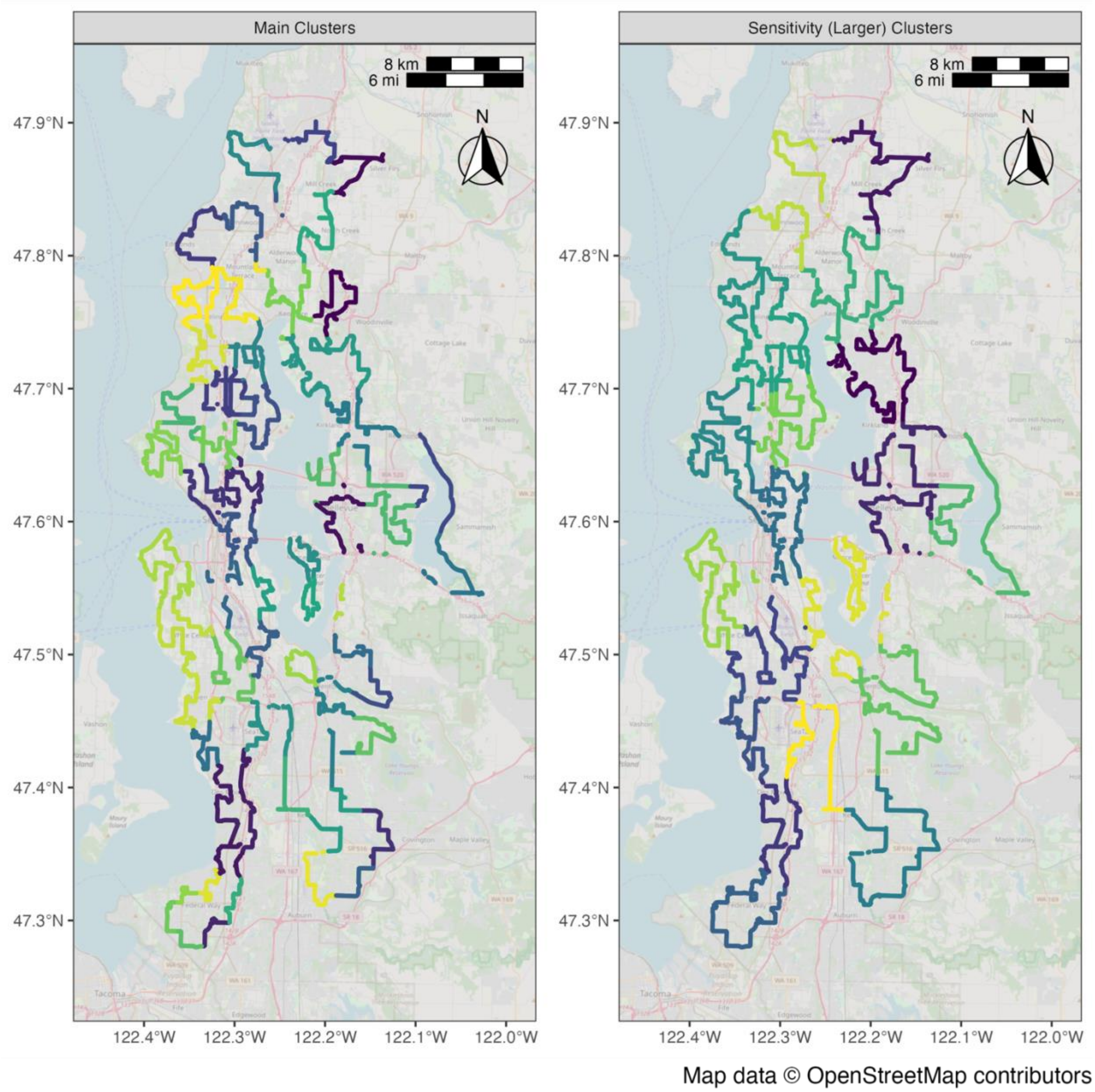

Figure S1. Spatial clusters used to conduct the unbalanced sampling designs. The main clusters (left) include an average of 93 100 m segments per cluster. Sensitivity clusters have approximately three times as many segments.

Note S1. Summary of temporal adjustment using an underwrite function approach

In summary, our temporal approach involved the following steps for each sampling campaign:

1. Simulating background PNC levels using the collected 1-second PNC measurements to calculate the first percentile of three-hour rolling averages.
2. Calculating hourly and long-term (~1 year) average background PNC.
3. Estimating hourly temporal adjustments based on the difference between hourly and long-term average background PNC.
4. Applying temporal adjustments to mobile monitoring visits conducted during business hours.
5. Estimating temporally adjusted annual average locations concentrations.

First, we simulated a continuous background PNC monitoring site using the collected PNC data since one was otherwise unavailable. We started with 3,359,505 unique 1-second on-road P-TRAK PNC measurements during the mobile monitoring campaign. To reduce the influence of extreme and potentially less reliable readings, we winsorized these measurement by setting measurements below the 5th percentile and above the 95th percentile to those respective thresholds. Additionally, we excluded measurements taken on highways (A1 roads) since they are less likely to represent background concentrations. This processing resulted in 2,978,809 (89%) 1-second measurements used to estimate background PNC over time, defined as the first percentile concentration of three-hour rolling windows (see Figure S2). Rolling windows were centrally aligned (e.g., for a three-hour window, data from 1.5 hours before and after were used). In sensitivity analyses, we evaluated different quantile-window combinations and included highway readings.

We used the resulting 1-second PNC background estimates, x_s (where $s = \{1, \dots, 2{,}978{,}809\}$) during the study period (~1 year) to calculate: (a) background PNC levels for each sampled hour h (where $h = \{1, \dots, 1{,}571\}$), and (b) a long-term average (LTA) background PNC (μ_{LTA}; Figure S3):

$$\mu_{\mathrm{h}} = \frac{1}{S}\sum_{s=1}^{S} x_{s,h} \quad \text{(S1)}$$

$$\mu_{\mathrm{LTA}} = \frac{1}{S}\sum_{s=1}^{S} x_{s} \quad \text{(S2)}$$

We then calculated hourly adjustment factors, δ_h, defined as the difference between the long-term average and hourly averages (see Table S1 for distribution of adjustments):

$$\delta_h = \mu_{LTA} - \mu_h \quad \text{(S3)}$$

These temporal adjustments were applied to adjust PNC measurements based on the exact time of sampling, allowing us to calculate temporally adjusted annual average PNC for each road segment. The temporally adjusted PNC for each visit v and location l is given by:

$$PNC_{v,l,\delta} = PNC_{v,l} + \delta_h \quad \text{(S4)}$$

The temporally adjusted long-term average PNC for each location l is then:

$$PNC_{LTA,l,\delta} \quad = \sum_{v=1}^{V_l} PNC_{v,l,\delta} \quad \text{(S5)}$$

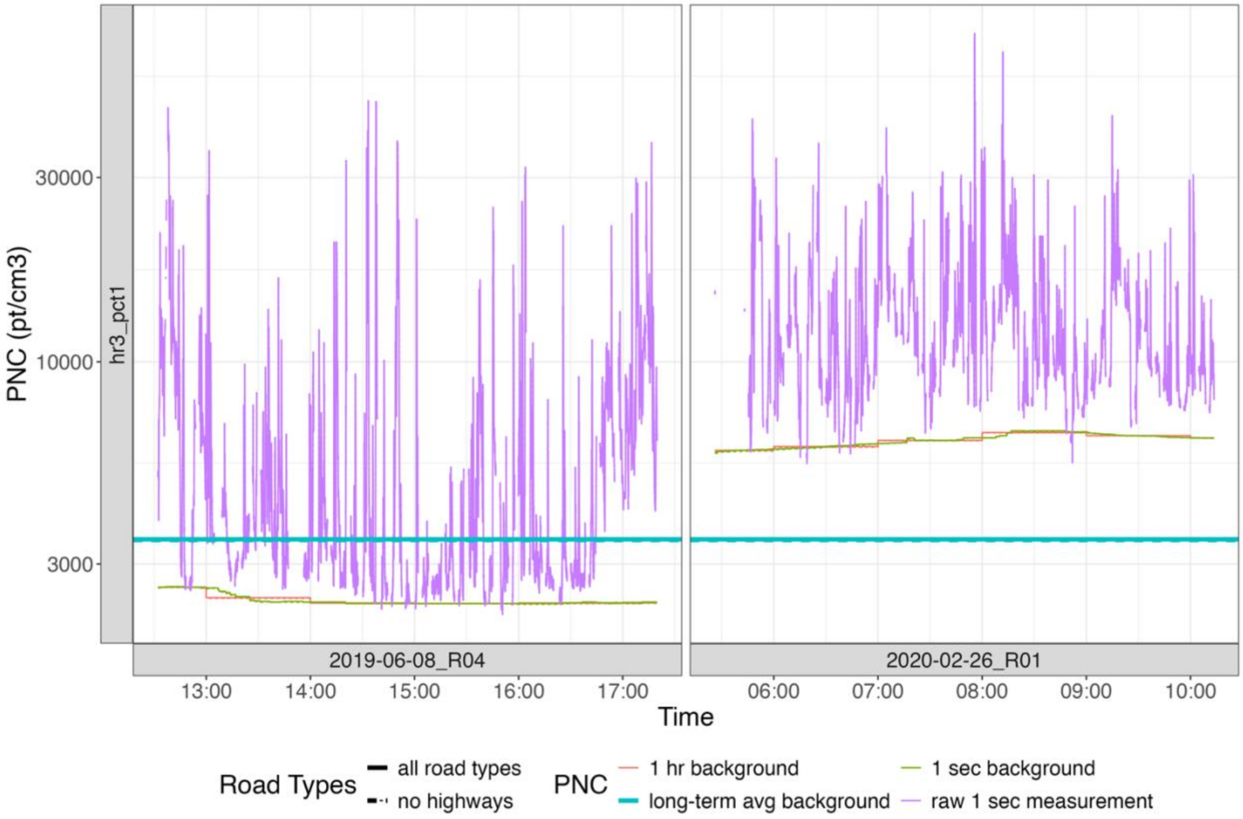

Figure S2. Example time series of measured and simulated background PNC for two sampling days. One-second P-TRAK field measurements are shown in purple. Background PNC is calculated as the 1st percentile of three-hour rolling windows (green); these values are used to estimate hourly PNC (red) and long-term average PNC (teal). The primary analysis focuses on non-highway measurements, while sensitivity analyses incorporate all road types, represented by different line styles.

(a)

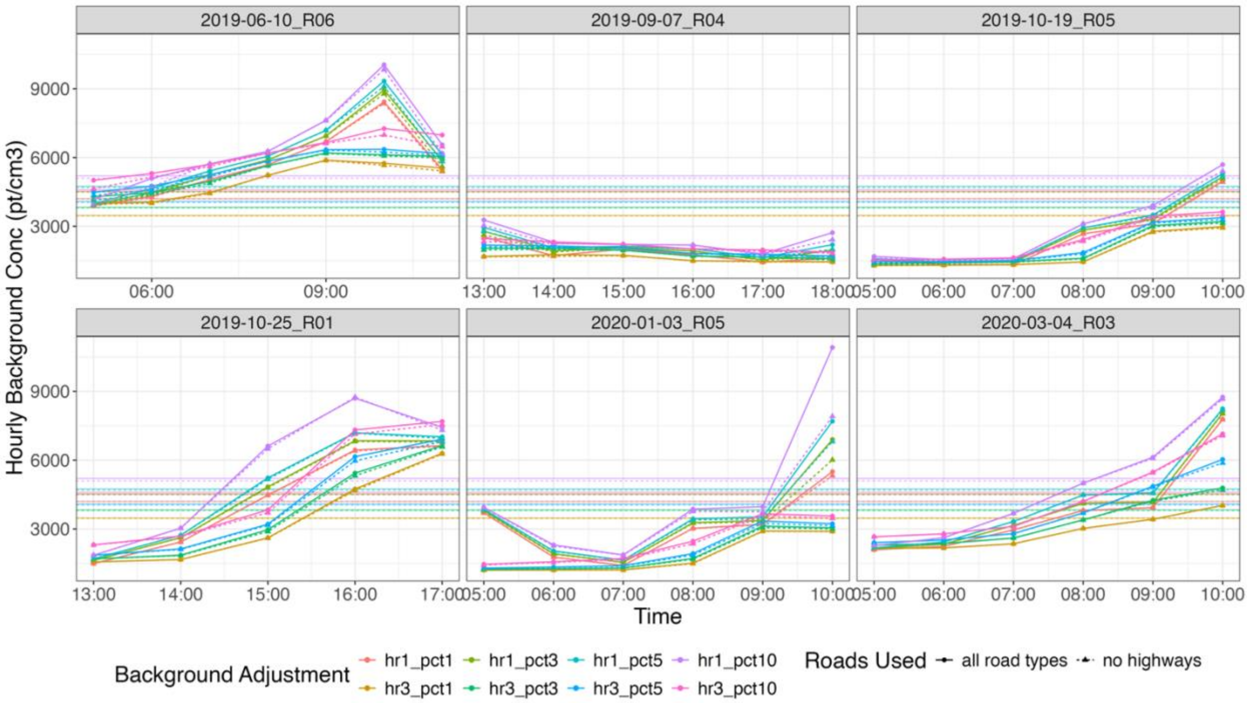

(b)

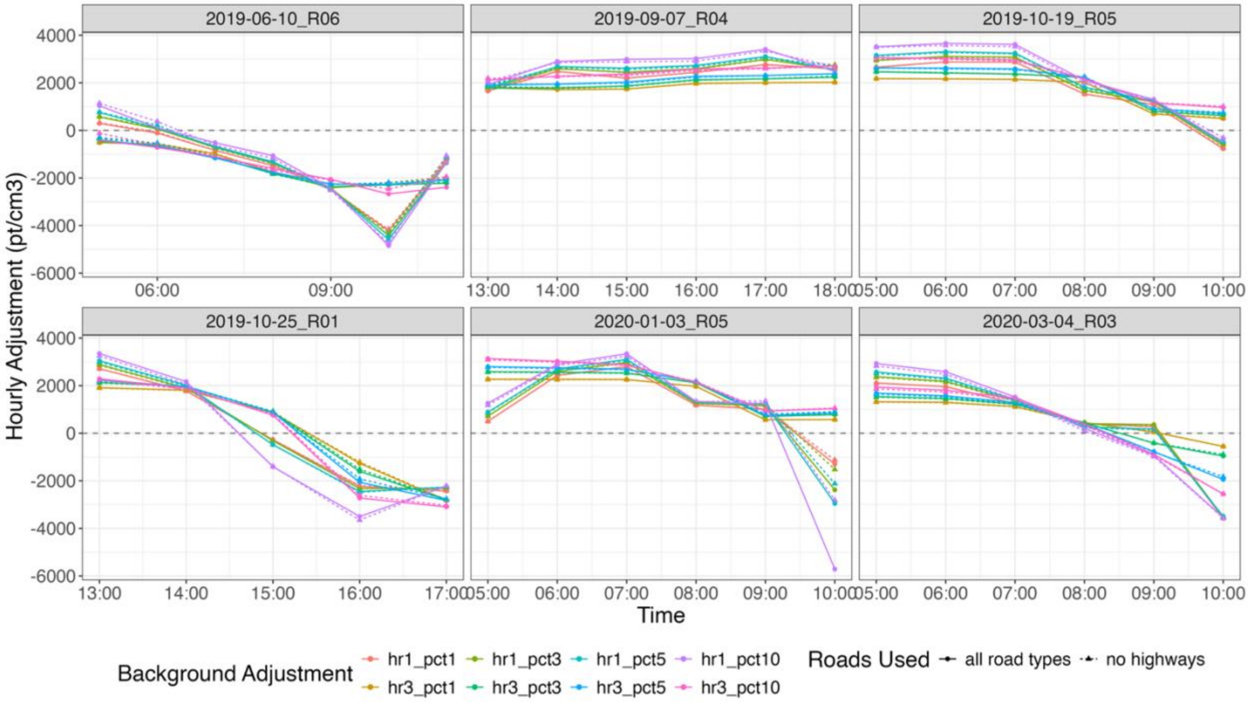

Figure S3. Example of temporal adjustments in sensitivity analyses evaluating different percentiles (1st to 10th) and time windows (1 to 3 hours) to define underwrite functions. (a) The top two rows show estimated hourly background concentrations and long-term averages (horizontal lines) for different sampling days. (b) The bottom two rows show the corresponding temporal adjustments. Different road types ("all road types" vs. "no highways") are represented by different line types. Despite variations in time windows, percentiles, and road types, results are highly consistent across all scenarios.

Table S1. Distribution of hourly P-TRAK PNC (pt/cm³) temporal adjustments applied to adjust business hours campaign visits.

Adjustment	Hours	Min	Q25	Median	Mean	Q75	Max	SD	IQR
Fixed Site	9,358	-5,592	-1,406	641	131	2,145	3,544	2,537	3,550
Underwrite	1,571	-11,387	-1,087	484	-53	1,422	2,972	2,054	2,509

2 Results

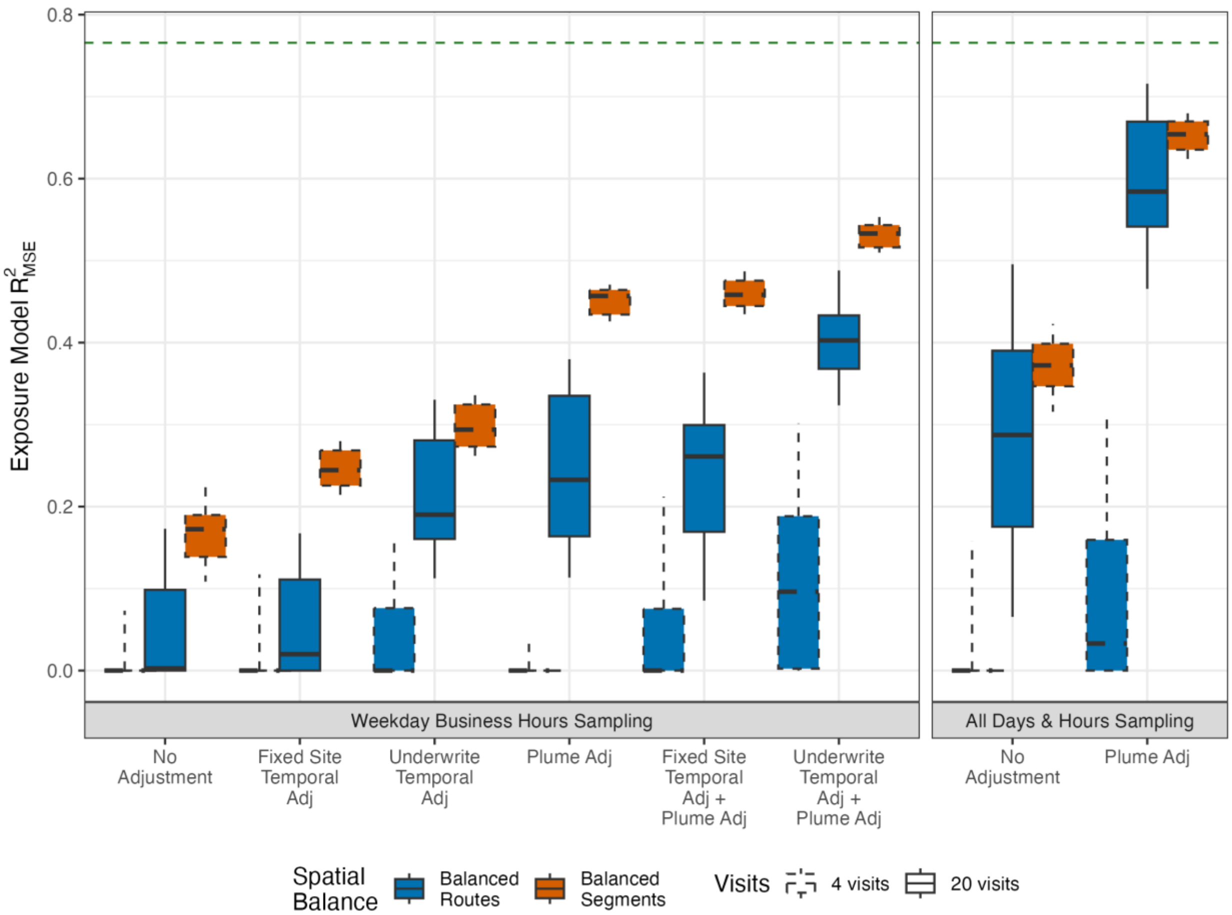

Figure S4. Out-of-sample PNC (pt/cm³) exposure model performances for on-road campaigns (N=30 campaigns per combination - i.e., boxplot) when sampling at the segment- vs. route-level. Compared to analyses that sample 4 visits per location at the segment level, analyses that consider logistical field constraints and sample entire routes generally perform worse, even when locations are visited 20 times (5 times as much). R^2_{MSE} is based on a comparison of the predicted PNC at 309 roadside locations to the annual average site estimates at those locations from stationary measures. The boxes illustrate the median and IQR, while the whiskers extend to the 10th and 90th percentiles. The dashed line shows the performance of the stationary roadside model.

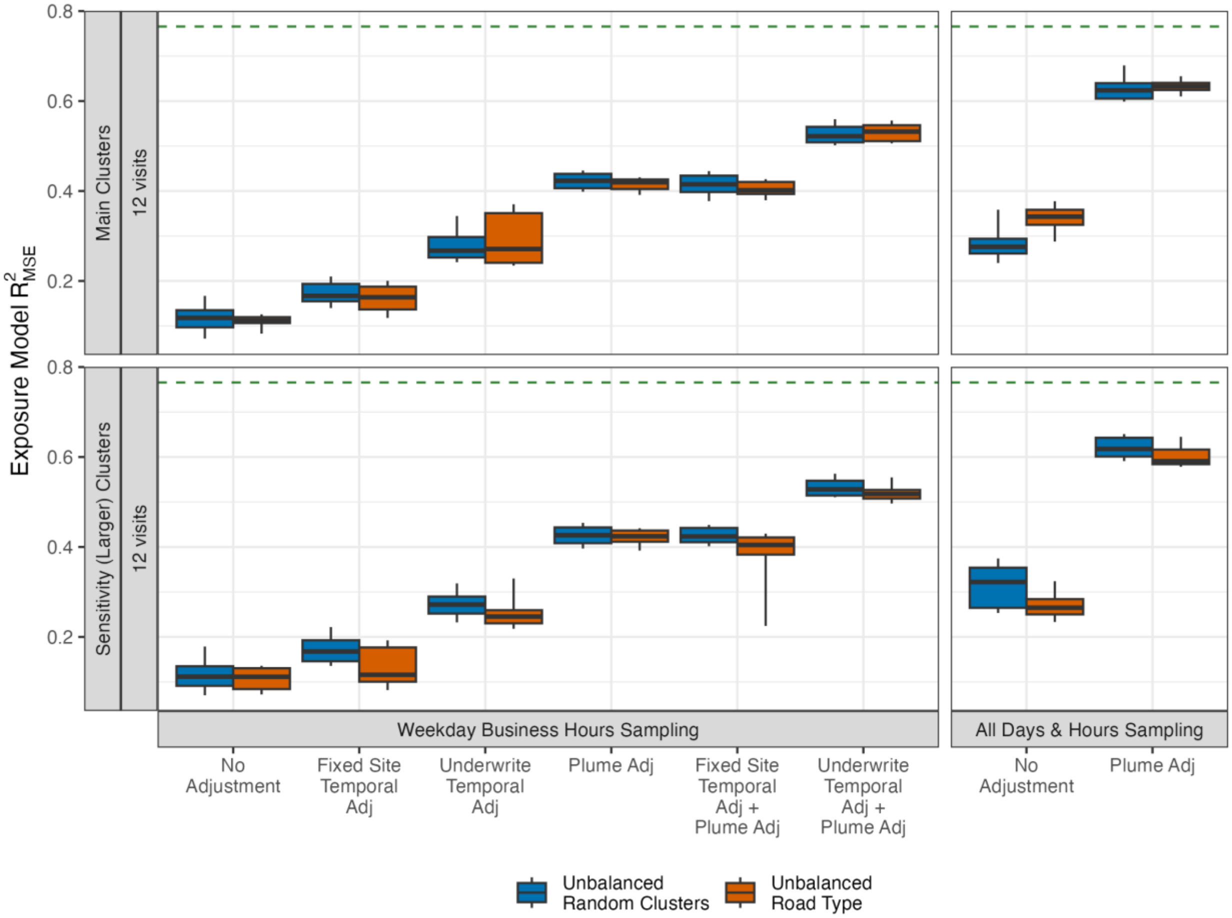

Figure S5. Out-of-sample PNC (pt/cm³) exposure model performances for on-road campaigns (N=30 campaigns per combination - i.e., boxplot) based on spatial clusters used in the main analysis and alternative, larger clusters. Results are for campaigns with 12 visits per location. R^2_{MSE} is based on a comparison of the predicted PNC at 309 roadside locations to the annual average site estimates at those locations from stationary measures. The boxes illustrate the median and IQR, while the whiskers extend to the 10th and 90th percentiles. The dashed line shows the performance of the stationary roadside model.

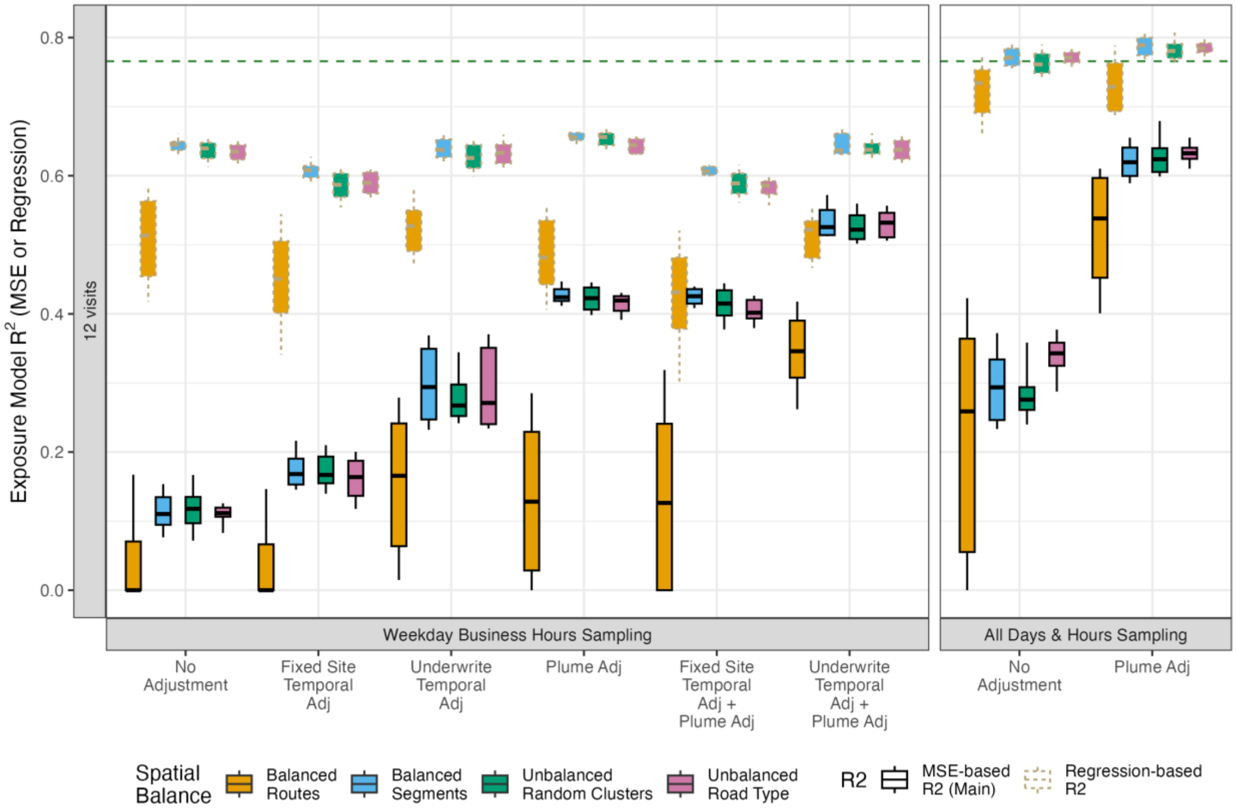

Figure S6. Out-of-sample PNC (pt/cm³) exposure model performances for on-road campaigns (N=30 campaigns per combination - i.e., boxplot) evaluated using R^2_{reg} for 12-visit campaigns. R^2_{reg} is based on a comparison of the predicted PNC at 309 stationary locations and the annual average site estimate based on stationary measures. The boxes illustrate the median and IQR, while the whiskers extend to the 10th and 90th percentiles. The dashed line shows the performance of the stationary roadside model for respective R², which is the same for R^2_{MSE} and R^2_{reg}.

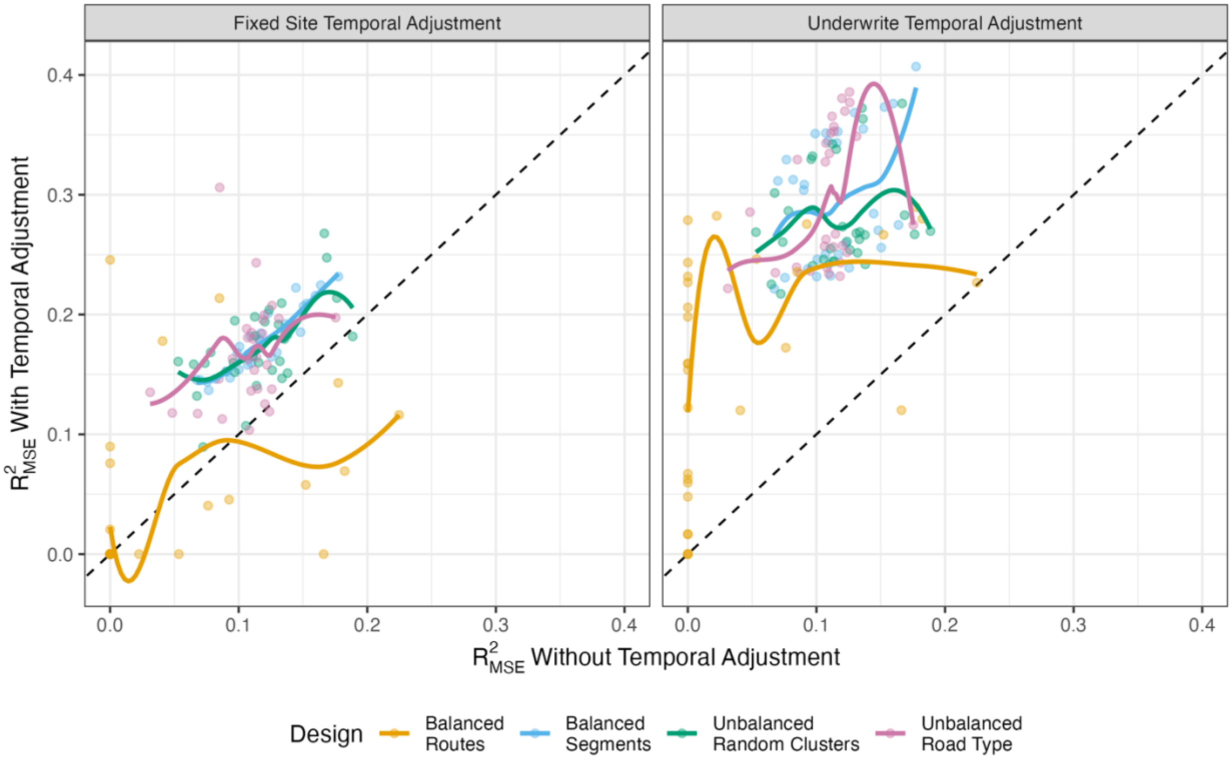

Figure S7. Comparison of R^2_{MSE} for a subset of 12-visit business hours sampling designs with and without temporal adjustment. The diagonal dashed line is the 1-1 line; values above this line indicate better resulting R^2_{MSE} values when compared to unadjusted campaigns.

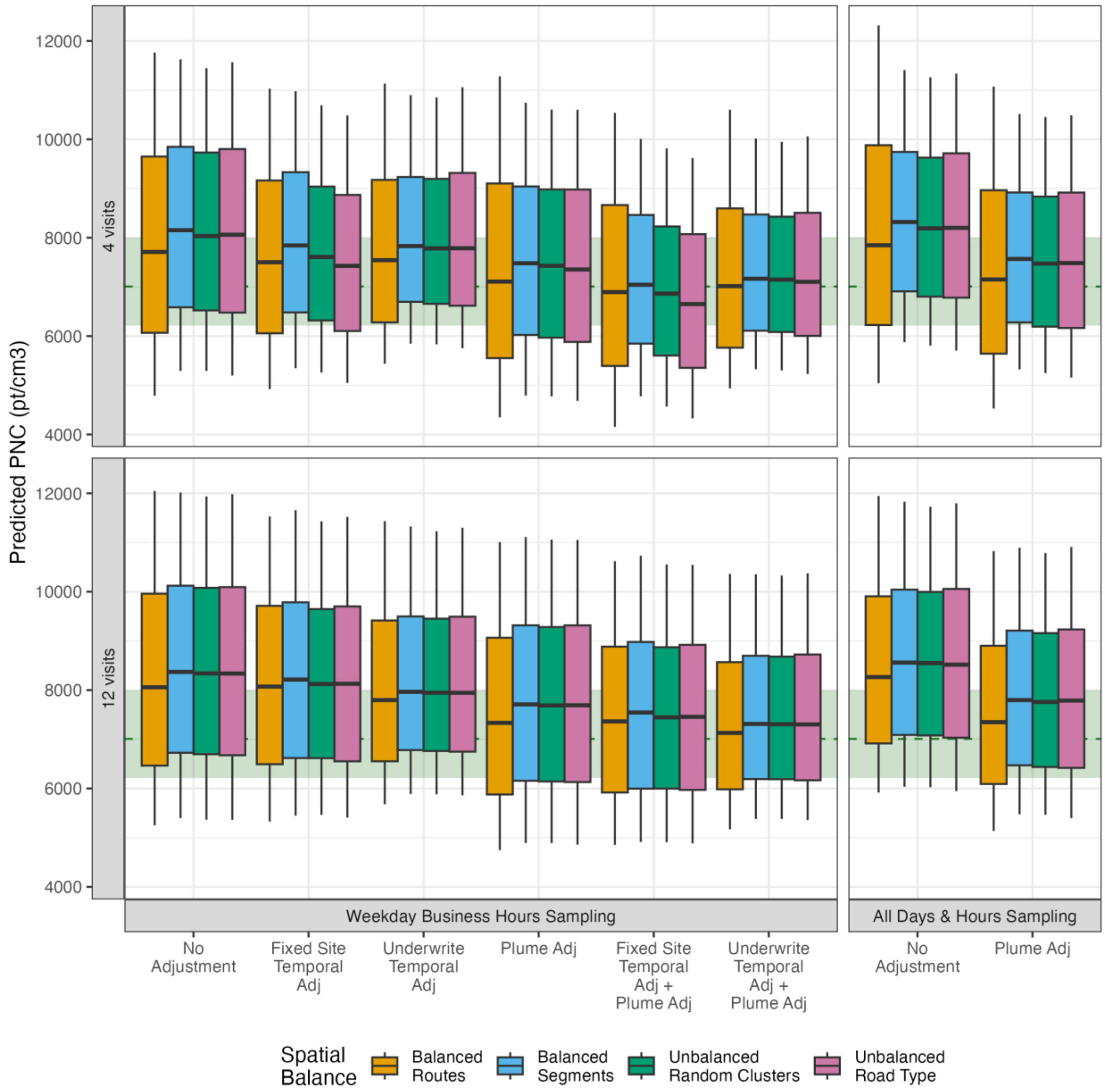

Figure S8. Predicted five-year average PNC exposure for ACT participants across 30 sampling campaigns for each design (i.e., boxplot). The green dashed line and area represent the median and IQR of cross-validated PNC predictions from the reference all-data stationary exposure model. The boxes illustrate the median and IQR, while the whiskers extend to the 10th and 90th percentiles.

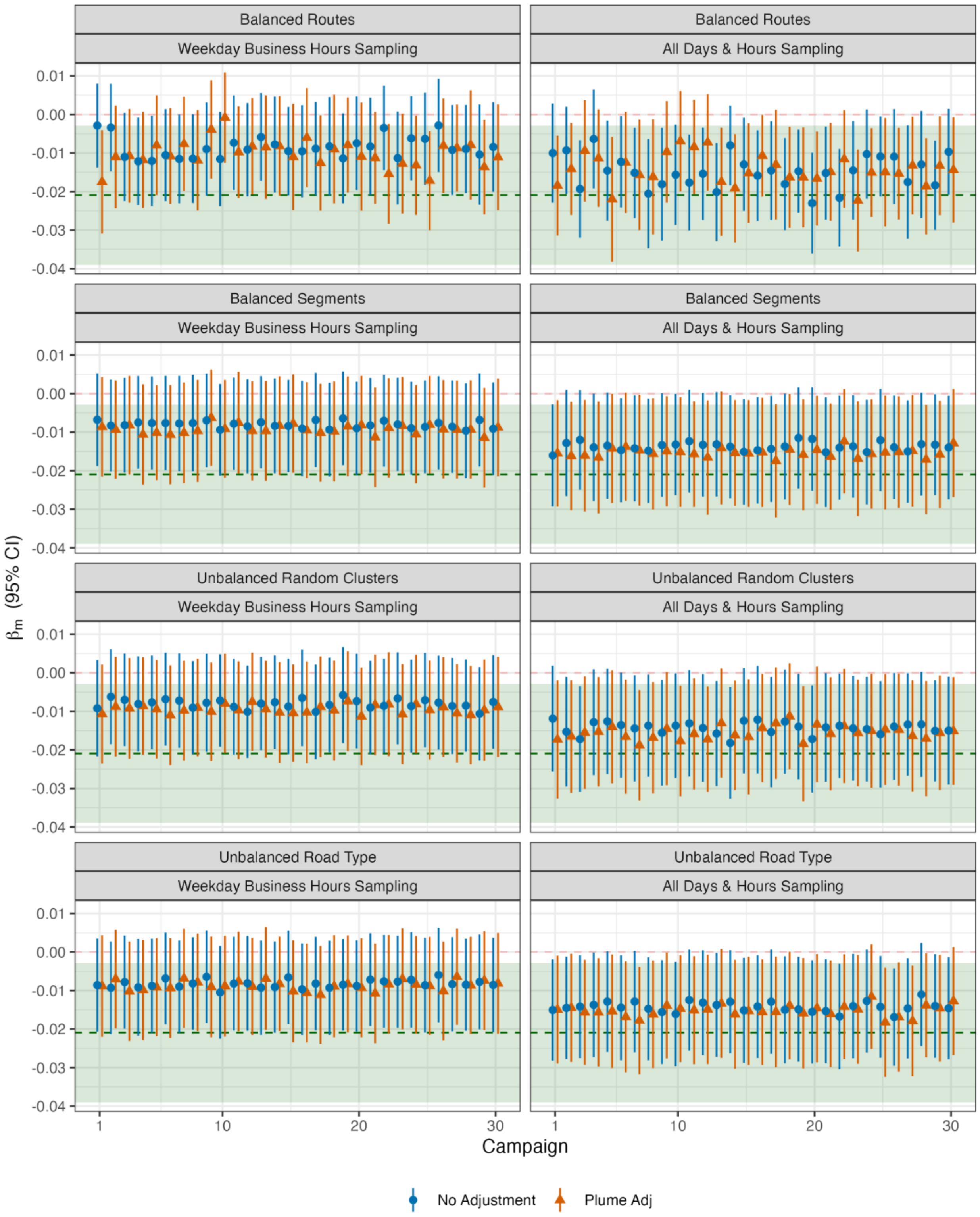

Figure S9. Point estimates and 95% confidence intervals (CI) from reduced health models of the adjusted association between PNC (1,900 pt/cm³) and CASI-IRT for selected campaigns. The green line and shaded area represent the point estimate and 95% CI from the roadside all-data exposure model, while the red line indicates no association.

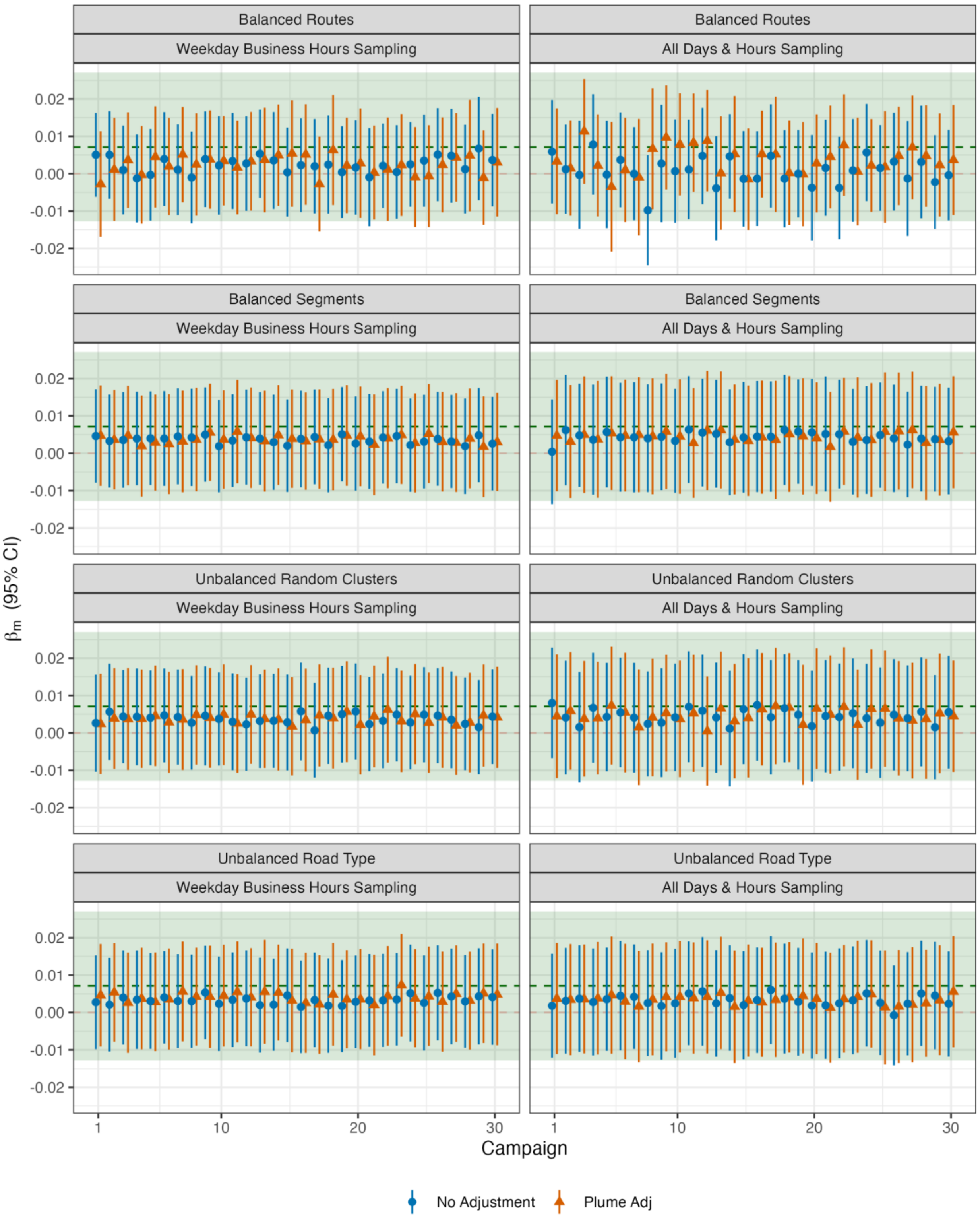

Figure S10. Point estimates and 95% confidence intervals (CI) from primary health models of the adjusted association between PNC (1,900 pt/cm³) and CASI-IRT for selected campaigns. The green line and shaded area represent the point estimate and 95% CI from the roadside all-data exposure model, while the red line indicates no association.

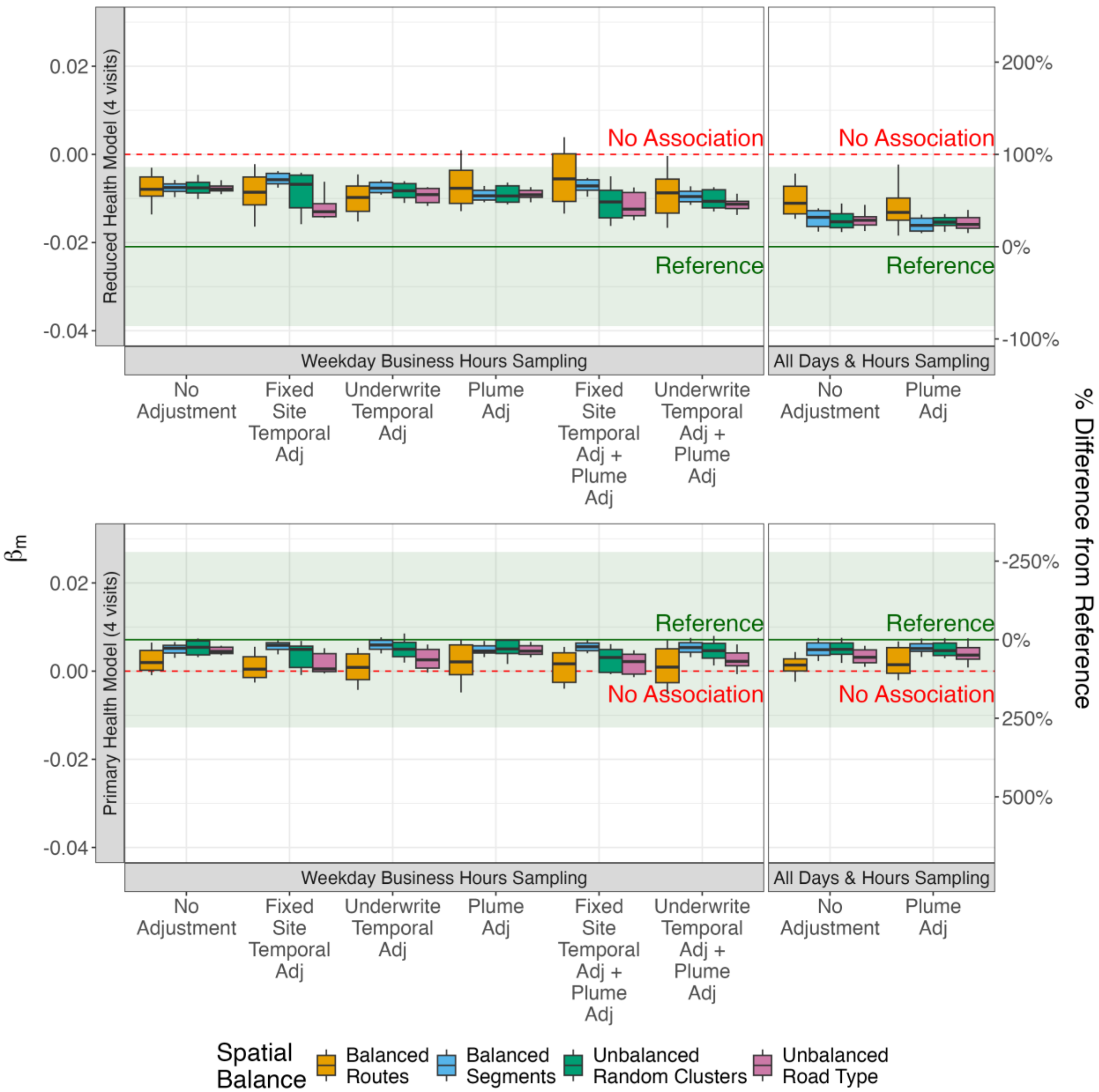

Figure S11. Estimated association between cognitive function (CASI-IRT) and PNC exposure (per 1,900 pt/cm³) for 4-visit campaigns. The dashed green line and colored area represent the reference health estimate and 95% CI from the roadside exposure model. The dashed red line indicates no association. Boxes show the median and IQR; whiskers illustrate the 10th and 90th percentiles of on-road campaign point estimates.
